# Effects of iso‐α‐acids, the hop‐derived bitter components in beer, on the MRI‐based Brain Healthcare Quotient in healthy middle‐aged to older adults

**DOI:** 10.1002/npr2.12077

**Published:** 2019-10-06

**Authors:** Masahiro Kita, Satoshi Yoshida, Keiji Kondo, Yoshinori Yamakawa, Yasuhisa Ano

**Affiliations:** ^1^ Research Laboratories for Health Science & Food Technologies Kirin Company Limited Yokohama Japan; ^2^ ImPACT Program of Council for Science Technology and Innovation (Cabinet Office, Government of Japan) Tokyo Japan

**Keywords:** bitter components, brain structure, fractional anisotropy, gray matter volume, iso‐α‐acids

## Abstract

**Aim:**

Neurological disorders are a major public health issue worldwide and are often associated with structural changes in the brain. We have previously demonstrated that iso‐α‐acids (IAAs), the hop‐derived bitter components in beer, improve memory impairment in aged and Alzheimer's disease mouse models. In this study, we evaluated the effects of IAA intake on the brain structure in healthy middle‐aged to older adults. This study was conducted under the Impulsing Paradigm Change through Disruptive Technologies Program (ImPACT) study launched by the Cabinet office of Japan.

**Method:**

This study employed an open‐labeled, single‐arm, before and after design. Healthy middle‐aged to older adults consumed a beverage containing IAAs (3 mg/190 mL) for 4 weeks.Recently developed magnetic resonance imaging‐based brain health indicators were used to evaluate the following brain conditions: the Brain Healthcare Quotient (BHQ) based on gray matter volume (GM‐BHQ) and white matter fractional anisotropy (FA‐BHQ).

**Results:**

In total, 25 subjects were recruited, and GM‐BHQ and FA‐BHQ were measured before and after intervention. In all subjects, no significant differences in GM‐BHQ and FA‐BHQ were observed. In subjects aged ≥ 60 years (mean 54.5; standard deviation 3.9) (n = 8), GM‐BHQ was significantly increased 4 weeks after intervention compared with that before intervention.

**Conclusion:**

Intake of beverages containing IAAs might affect brain aging, particularly in healthy older adults, which may prevent the development of neurological disorders. Future studies employing more robust designs can elucidate the effects of IAAs on GM‐BHQ and cognitive functions.

## Abstract 2

This is the first clinical trial evaluating the effects of intake of bitter component of beer, iso‐alpha‐acid, on brain structure. The brain structure before and after intervention was measured by recently developed magnetic resonance imaging‐based method. The gray matter volume of older adults was improved by the intake of iso‐alpha‐acid.

## Graphical abstract

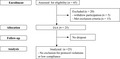

## 1 INTRODUCTION

With an aging society, the number of patients exhibiting neurological disorders has rapidly increased worldwide, which is burden on the patients and society.^1^ Maintenance of brain health and prevention of neurological disorders are important aspects required to decrease social costs. Disease pathologies of neurological disorders, including Alzheimer's and Parkinson's diseases, are associated with structural changes in the brain, primarily atrophy.^2^ Brain structure atrophy is associated with negative neurological symptoms, such as cognitive decline.^3^ Thus, maintaining a healthy brain structure is important for the prevention of various neurological disorders, and structural brain changes are a useful indicator for the health status of the brain. Daily food‐derived nutrients have acquired increasing attention as a preventive approach for neurological diseases.

The herb hop (*Humulus lupulus* L.) is a well‐known brewing material and is a primary ingredient in beer. Hops have long been used in brewing to add bitterness and flavor. Iso‐α‐acids (IAAs) are the main bitter components in beer produced from hops. Commercially available beer typically contains IAAs at 10‐30 ppm; furthermore, nonalcoholic beverages simulating beer contain IAAs. Our group has previously demonstrated that IAAs suppress brain inflammation and prevent cognitive impairment in Alzheimer's transgenic and aged mice.^4^ In aged mice, IAA intake improved hippocampal atrophy and memory impairment. In addition, we recently revealed that IAAs activate the vagal nerve and improve memory function in mice through the dopamine system.^5^ Overall, IAA intake is expected to improve or maintain brain function. However, no studies have evaluated the effects of IAA intake on brain health in humans.

Recently, the Impulsing Paradigm Change through Disruptive Technologies Program (ImPACT) initiated by the Japanese cabinet office has developed the Brain Healthcare Quotient (BHQ) based on gray matter (GM) volume (GM‐BHQ) and the white matter fractional anisotropy (FA; FA‐BHQ) as the standardized health index of brain structure in healthy individuals using magnetic resonance imaging (MRI).^6^ (GM‐BHQ and FA‐BHQ of each subjects were shown in Table S1). The GM volume reflects the number of dendrite expanse and neural cell synapses,^7^ and the white matter FA that is measured using diffusion tensor imaging (DTI) reflects the plasticity of white matter and the efficacy of neural transmissions in the brain.^8^ In an ImPACT study, GM‐BHQ and FA‐BHQ have been developed and standardized based on the MRI images of approximately 150 healthy subjects. These indexes are simple and are highly correlated with aging and various physical and social factors (eg, body mass index or subjective well‐being, respectively) that are associated with the development of neurological disorders. Thus, these can be useful indicators of brain health in healthy subjects. Preventative approaches improving GM‐BHQ and FA‐BHQ are required to maintain brain health and prevent neurological disorders.

This study is the first clinical trial evaluating the effects of IAAs on human brain health as indicated by GM‐BHQ and FA‐BHQ. We recruited 25 healthy middle‐aged to older adults, and they consumed beverages containing 3.4 mg of IAAs each day for 4 weeks. This study employed an open‐labeled, single‐arm, before and after design and was conducted under the ImPACT study.

## 2 METHODS

### 2.1 Subjects

We recruited 45 right‐handed healthy Japanese adults, aged 50‐70 years, who were not habitual alcohol drinkers (ie, consuming alcohol less than once a week). Exclusion criteria were listed on Table 1. At enrollment, inclusion and exclusion criteria were examined using a questionnaire.

**Table 1:** The list of exclusion criteria for this study

No.	Excursion criteria
1.	Smoking
2.	History of severe heart, liver, or kidney disease
3.	Regular drug treatment
4.	Treatment with dietetics or kinesitherapy
5.	Medicinal or food allergies
6.	History of drug abuse
7.	Irregular lifestyle such as shift work
8.	Pregnancy, lactation, or planning pregnancy
9.	Participation in other clinical studies
10.	Use of artificial cardiac pacemakers
11.	Use of vascular clips
12.	Use of electrical stimulator for nerves
13.	Use of embedded pumps
14.	Metal in the body because of accidents or injuries
15.	Tattoos
16.	Metal or screws in the bodies because of treatment for broken bones
17.	Use of dyes containing metals to color hair
18.	Use of glycerin patches
19.	Use of artifacts in the ears
20.	Eye injuries caused by metals
21.	History of injury because of surgery
22.	Use of intrauterine devices
23.	Use of dental instruments containing metals,
24.	Artificial eyes
25.	History of labyrinthitis disease such as Meniere's disease
26.	Use of artificial cardiac valves
27.	Claustrophobia or nyctophobia
28.	Dyskinesia
29.	Severe visual disorder(s) or hearing impairments
30.	History of heart seizures
31.	History of epileptic seizures
32.	Migraines
33.	Sensitivity to lights
34.	Occupational history of polishing metals

### 2.2 Experimental supplements

In hops, α‐acids predominantly consist of three congeners: cohumulone, humulone, and adhumulone. During the brewing process, these are isomerized into two epimeric isomers, namely cis‐ and trans‐IAAs. In the present study, we used isomerized hop extract (Hopsteiner) with 30.5% (w/v) IAAs, comprised of trans‐isocohumulone (1.74% w/v), cis‐isocohumulone (7.61% w/v), trans‐isohumulone (3.05% w/v), cis‐isohumulone (14.0% w/v), trans‐isoadhumulone (0.737% w/v), and cis‐isoadhumulone (3.37% w/v) as previously described.^9^

The test formula consisted of a 190‐mL beverage containing isomerized hop extract and containing 3.4‐mg IAAs; this beverage was ingested every day for 4 weeks, and it contained acidulant, sweetener, and flavor to mask the bitterness of IAAs. The IAA dose in this study is approximately equivalent to the IAA content in 500 mL of commercially available beer.

### 2.3 Procedures

This study was performed using an open‐labeled, single‐arm, before and after design. Questionnaires for the inclusion/exclusion criteria were answered at enrollment. All eligible subjects consumed the test beverage for 4 weeks, and subjects were not blinded to the information regarding the test beverage. Subjects were instructed to maintain their regular lifestyles and avoid consumption of any foods or beverages containing hops. Compliance was monitored using subject diaries. MRI measurements were obtained before and after 4 weeks of beverage intake. On the MRI measurement day, subjects were instructed to completely avoid food and beverages containing caffeine. The data were collected at the University of Tokyo (Tokyo, Japan) between November 2015 and December 2015.

### 2.4 GM‐BHQ and FA‐BHQ measurements

High‐resolution MRI image acquisition and GM‐BHQ and FA‐BHQ calculations were performed according to previously described methods.^6^ A 3‐T Siemens scanner (Verio, Siemens Medical Solutions; or MAGNETOM Prisma, Siemens) with a 32‐channel head array coil was used for MRI image acquisition. A three‐dimensional T1‐weighted magnetization‐prepared rapid acquisition gradient echo pulse sequence was used for high‐resolution structural image acquisition. DTI data were acquired using spin‐echo echo‐planar imaging with generalized autocalibrating partially parallel acquisitions. Statistical Parametric Mapping 12 (Wellcome Trust Centre for Neuroimaging) running on MATLAB R2015b (Mathworks Inc) was employed for processing and analyzing T1‐weighted MRI images. Individual GM‐BHQ and FA‐BHQ were calculated using the following formula: 100 + 15 × (individual GM or FA − mean)/SD. Mean and SD were employed from the previous study.^6^

### 2.5 Statistics

The Kolmogorov‐Smirnov test was performed in order to check the normality of the data set. Then, changes in GM‐BHQ and FA‐BHQ were analyzed by a paired *t* test. All statistics were performed using Ekuseru‐Toukei 2015 (Social Survey Research Information Co Ltd).

## 3 RESULTS

### 3.1 Subject characteristics

**Figure 1:**
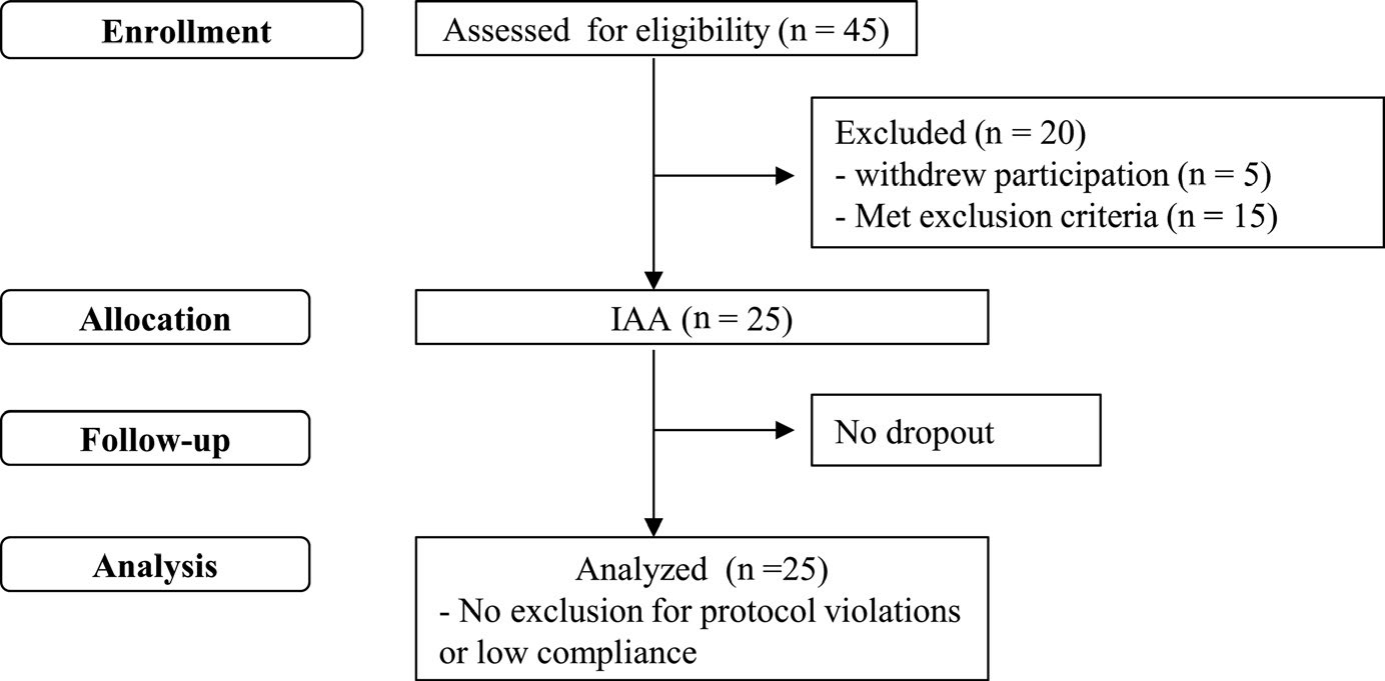
The flow of the subjects. In total, 45 subjects were assessed for eligibility, and 20 subjects were excluded. The remaining 25 subjects consumed the beverage containing IAAs for 4 wks. All subjects completed the schedule, and all 25 subjects were included for analysis

The flow of subjects through this study is shown in Figure 1. Following the inclusion criteria, 20 subjects were excluded because of participation withdrawal (n = 5) or exclusion criteria fulfillment (n = 15). The remaining 25 subjects consumed beverages containing IAAs for 4 weeks. No subjects withdrew from participation during this intervention. In conclusion, 25 subjects completed the study, and none were excluded from the final analysis. The subject characteristics are shown in Table 2.

**Table 2:** Subject characteristics at baseline

Characteristics	IAA
Age	57.4 ± 6.1
Male/Female	14/11

Note

Data are the mean ± SD (n = 25).

Abbreviation: IAA, iso‐α‐acids.

### 3.2 Changes in GM‐BHQ and FA‐BHQ

**Figure 2:**
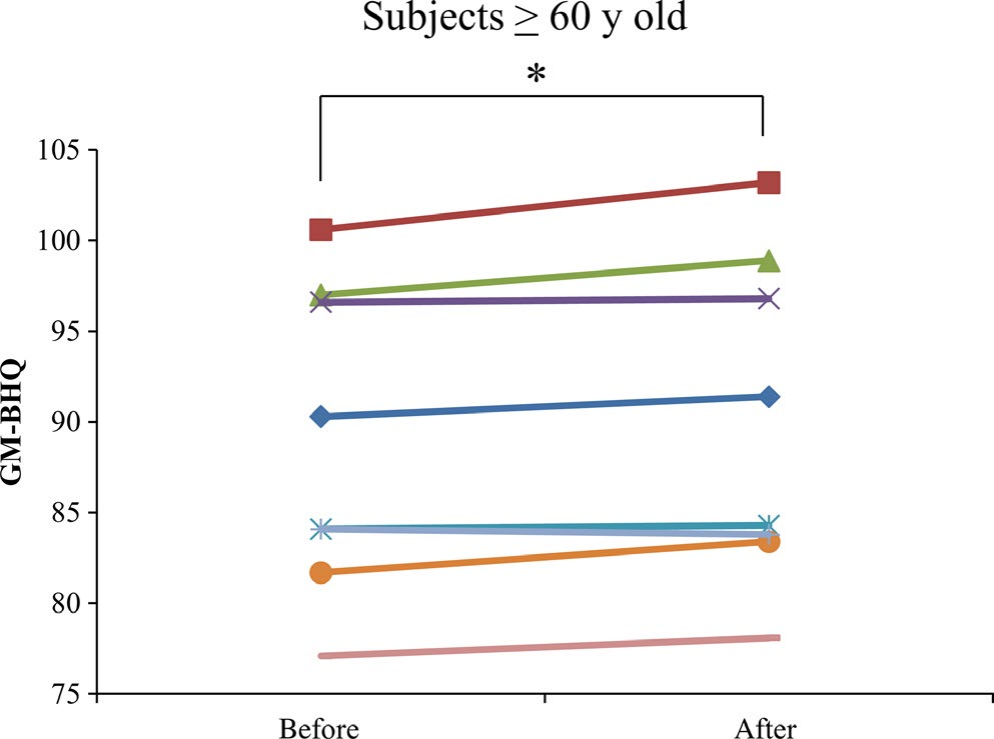
Changes in GM‐BHQ in subjects ≥60 y of age. The each line shows the changes in GM‐BHQ in subjects ≥60 y of age. Data represent the changes in each subject. *shows the *P* < .05 performed by paired *t* test. GM‐BHQ, the Brain Healthcare Quotient based on gray matter volume

Changes in GM‐BHQ and FA‐BHQ before and after intervention are shown in Table 3; no significant differences were observed. GM‐BHQ and FA‐BHQ were previously reported to be sensitive to aging.^4^ Thus, we classified subjects into two groups, <60 (54.5 ± 3.9, n = 17) or ≥60 (64.9 ± 3.3, n = 8) years of age, and evaluated the effects of IAAs in older subjects. Changes in GM‐BHQ and FA‐BHQ among subjects classified according to age are shown in Table 3. GM‐BHQ, but not FA‐BHQ, was significantly increased after intervention compared with that before intervention in older subjects (Table 4, Figure 2). In younger subjects, GM‐BHQ and FA‐BHQ remained unchanged before and after intervention.

**Table 3:** Changes in GM‐BHQ and FA‐BHQ

	Before intervention	After intervention
GM‐BHQ	95.0 ± 7.6	94.9 ± 7.6
FA‐BHQ	97.8 ± 3.6	97.6 ± 3.3

Note

Data are the mean ± SD.

Abbreviations: FA‐BHQ, the Brain Healthcare Quotient based on white matter fractional anisotropy; GM‐BHQ, the Brain Healthcare Quotient based on gray matter volume.

**Table 4:** Changes in GM‐BHQ and FA‐BHQ before and after intervention within subjects classified by age

	≥60 (n = 8)		<60 (n = 17)	
	Before	After	Before	After
GM‐BHQ	88.9 ± 8.5	90.0 ± 8.4*	97.8 ± 5.1	97.8 ± 5.0
FA‐BHQ	96.4 ± 4.0	95.9 ± 3.4	98.5 ± 3.3	98.3 ± 3.1

Note

Data are the mean ± SD.

Abbreviations: FA‐BHQ, the Brain Healthcare Quotient based on white matter fractional anisotropy; GM‐BHQ, the Brain Healthcare Quotient based on gray matter volume.

* Shows *P* < .05 performed by paired *t*‐test.

## 4 DISCUSSION

In this study, we first evaluated the effects of IAAs on brain health in humans. GM‐BHQ, but not FA‐BHQ, significantly increased following IAA intake, particularly in older subjects. GM‐BHQ reflects the volume of GM associated with an expanse of dendrites, whereas FA‐BHQ is associated with white matter plasticity.^7^, ^8^ Thus, IAA intake may improve brain health by affecting GM volume.

GM‐BHQ is reported to decrease with age.^6^ Therefore, older subjects were predicted to be more sensitive to IAA intake. Amyloid‐β (Aβ) accumulation and inflammation in the brain increases with age, even in subjects without symptoms of neurological disorders.^10^, ^11^ These events damage neural cells, resulting in a decrease in GM volume.^10^, ^12^ IAA intake has been reported to suppress inflammation and Aβ accumulation in the hippocampus and frontal cortex in Alzheimer's disease transgenic and aged mouse models.^4^ A decrease in hippocampal weight in aged mice was suppressed by IAA intake (unpublished data). IAA intake at 1 mg/kg for 7 days was shown to reduce microglial inflammation and improve neural hyperactivation, which was detected using manganese MRI in an Alzheimer's disease mouse model.^4^ In addition, IAAs have been reported to activate the peroxisome proliferator‐activated receptor (PPAR)‐γ.^13^ Low‐dose administration of the PPAR‐γ agonist pioglitazone improved resting‐state functional connectivity in rat brains.^14^ These reports suggest that the suppression of inflammation by IAAs through PPAR‐γ may be associated with GM‐BHQ improvement.

Furthermore, GM‐BHQ exhibits a negative correlation with body mass index.^6^ Obesity and obesity‐associated metabolic disorders such as insulin resistance are strong exacerbating factors of dementia.^15^ In addition, an association between obesity and brain atrophy has been reported.^16^ Obesity exacerbates brain inflammation, resulting in neural cell damage.^15^ IAA intake has been reported to improve metabolic syndromes, including obesity 17, 18, 19; obesity‐induced cerebral inflammation; brain atrophy; and cognitive decline in a high‐fat diet‐induced obesity mouse model.^20^ In the clinical trial, IAA intake at 16 mg/d for 4 weeks resulted in decreased HbA1c levels, and IAA intake at 48 mg/d for 12 weeks resulted in decreased body mass indexes in subjects with prediabetes.^19^ Thus, IAAs may be associated with improved body metabolism and increased GM‐BHQ. However, we could not obtain metabolic factors in this study, so we could not assert that the improvement of GM‐BHQ by the intake of IAAs was due to the improvement of metabolic conditions. Further study evaluating the relationship between the effects of IAAs on GM‐BHQ and metabolic factors are needed to elucidate the underlying mechanisms in detail.

In addition to cognitive decline or dementia, GM‐BHQ is affected by psychological conditions. It was reported that GM‐BHQ was declined in the group with the subjective fatigue, which is associated with fatigue and stress.^21^ Recently, it has been reported that psychological stress causes microglial activation, leading to the productions of inflammatory cytokines such as tumor necrosis factor‐α and interleukin‐6 in the brain 22. Excessive inflammatory cytokines damaged neural cells in GM, causing subjective fatigue, and finally leading to the psychological disorders including major depression and chronic fatigue syndrome.^22^, ^23^ Previously, our group has demonstrated that IAA suppressed the activation of microglia and the levels of inflammatory cytokines in the brain of lipopolysaccharide‐inoculated mice.^24^ Furthermore, hop‐derived bitter compounds of matured hop bitter acids also suppressed cerebral inflammation and ameliorated depression‐like behavior in lipopolysaccharide‐inoculated mice.^25^ Taken together, it is suggested that the intake of IAA suppressed microglial activation and cerebral inflammation, leading to the improvement of GM‐BHQ and psychological conditions.

This study has several limitations. First, an open‐labeled, single‐arm, before and after design was employed, which could not preclude potential placebo effects. GM‐BHQ and FA‐BHQ are objective indexes; therefore, the placebo effects may not have significantly influenced the results. However, a study employing a more robust design is required to increase the reliability of our conclusions. Second, we did not evaluate the effects of IAAs on cognitive function or mood status with neuropsychological methods; therefore, the effect of IAA intake on cognitive functions or mood by enhancing GM‐BHQ remains unclear. Future studies are required to clarify the association between these indexes and neurological disorders and elucidate the clinical significance associated with the improvement of these indexes.

In conclusion, IAA intake improves the decrease in GM‐BHQ associated with aging and contributes to brain health maintenance, which may prevent the development of neurological disorders. The IAA dose utilized in this study is easily consumed daily in alcoholic or nonalcoholic beverages. Thus, IAA intake could be a practical method to maintain brain health.

## CONFLICT OF INTEREST

M.K., S.Y., K.K., and Y.A. are employed by the funder, and Y.Y. is the member of ImPACT. The test beverage was prepared by the funder. The MRI data collection and analysis were performed by ImPACT.

## AUTHOR CONTRIBUTIONS

M.K. and Y.A. designed and conducted the study. S.Y. and K.K. supervised the study. Y.Y. conducted this study as ImPACT manager. M.K. wrote the manuscript.

## ETHICAL APPROVAL

The study was conducted in accordance with the Declaration of Helsinki and the recommendations of Ethical Guidelines for Medical and Health Research Involving Human Subjects, Ministry of Health, Labor and Welfare. This study was approved by ethics committee of Huma R&D CO. Ltd (Tokyo, Japan) and the University of Tokyo (Tokyo, Japan).

## DATA REPOSITORY

I agree to publish part of it as supplementary information.

## APPROVAL OF THE RESEARCH PROTOCOL BY AN INSTITUTIONAL REVIEWER BOARD

This study was approved by the ethics committee of Huma R&D Co Ltd. (Tokyo, Japan) and the University of Tokyo (Tokyo, Japan).

## INFORMED CONSENT

Written informed consent was obtained from all participants.

## REGISTRY AND THE REGISTRATION NO. OF THE STUDY

The study was registered on November 30, 2015, in the database of the University Hospital Medical Information Network (UMIN) (Registration No. UMIN000020006; Registration title: A study for the effect of intake of bitter component derived from hops on cognitive functions).

## ANIMAL STUDIES

n/a.
