# Supplementary material for: Effects of iso‐α‐acids, the hop‐derived bitter components in beer, on the MRI‐based Brain Healthcare Quotient in healthy middle‐aged to older adults
Source: Neuropsychopharmacol Rep. 2019 Oct 6;39(4):273–8. doi: 10.1002/npr2.12077 (PMC7292307; doi:10.1002/npr2.12077)
Supplement: Supplementary file 1 [file NPR2-39-273-s001.docx]

Supplementary table. 1 GM-BHQ and FA-BHQ of each subjects

|  |  | GM-BHQ |  | FA-BHQ |  |
| --- | --- | --- | --- | --- | --- |
| Age | Sex | Pre | Post | Pre | Post |
| 58 | M | 93.2 | 92.9 | 99 | 96.9 |
| 51 | F | 101.4 | 101.8 | 101.7 | 101.2 |
| 61 | M | 90.3 | 91.4 | 95 | 93 |
| 54 | F | 109 | 109 | 106.3 | 105.7 |
| 50 | F | 98.9 | 99.3 | 98.1 | 97.9 |
| 59 | F | 94.9 | 95.6 | 98.4 | 98.6 |
| 57 | F | 95.6 | 93.5 | 97.1 | 98.9 |
| 62 | F | 100.6 | 103.2 | 98.5 | 99.1 |
| 58 | M | 108 | 106.8 | 102.4 | 101.6 |
| 64 | F | 97 | 98.9 | 96.4 | 96.6 |
| 66 | M | 96.6 | 96.8 | 97.1 | 97 |
| 68 | M | 84.1 | 84.3 | 95.2 | 93.4 |
| 55 | M | 90.4 | 91.8 | 101.8 | 101.6 |
| 61 | M | 81.7 | 83.4 | 104.1 | 101.6 |
| 68 | F | 84.1 | 83.8 | 90 | 91 |
| 54 | M | 99.6 | 101.6 | 95.8 | 96.2 |
| 54 | F | 96.4 | 96.1 | 99.7 | 98.8 |
| 53 | M | 97.5 | 96.8 | 94.1 | 93.3 |
| 58 | M | 94.1 | 94.3 | 95.4 | 96.2 |
| 50 | F | 101.4 | 100.4 | 97.9 | 97.7 |
| 54 | M | 92.1 | 94 | 94.1 | 94.2 |
| 69 | M | 77.1 | 78.1 | 95.1 | 95.8 |
| 51 | M | 92.6 | 92.2 | 96.1 | 96 |
| 50 | F | 101.5 | 100.5 | 100.1 | 100.6 |
| 51 | M | 96.7 | 86.1 | 95.8 | 96 |

M: Male, F: Female
